# Editorial: Research in the identification and control methods of rot diseases in plants

**DOI:** 10.3389/fmicb.2025.1601422

**Published:** 2025-04-14

**Authors:** Mohammad Arif, Quan Zeng, Shefali Dobhal

**Affiliations:** ^1^Department of Plant and Environmental Protection Sciences, University of Hawaii at Manoa, Honolulu, HI, United States; ^2^Department of Plant Pathology and Ecology, Connecticut Agricultural Experiment Station, New Haven, CT, United States

**Keywords:** soft rot and blackleg disease, bacteria, fungi, *Dickeya*, *Pectobacteriaceae*, *Fusarium*

Rot is a major concern in plant agriculture, jeopardizing crop production, yield, storage, and overall quality of a wide variety of crops (Bolton et al., 2006; Dean et al., 2012; Mansfield et al., 2012; Boluk et al., 2021; Klair et al., 2021; Sinha et al.). It is caused by a diverse array of fungal and bacterial pathogens that infect plant tissues, leading to the degradation of structural integrity and eventually the decay of roots, stems, leaves, or harvested produce (Williamson et al., 2007; Boland and Hall, 1994; Bolton et al., 2006). These infections can occur at any stage of the plant life cycle, from seedling to post-harvest storage, significantly lowering marketability and contributing to substantial monetary loss globally (Dean et al., 2012; Mansfield et al., 2012). Fungal pathogens such as *Botrytis cinerea, Fusarium* spp., *Sclerotinia sclerotiorum*, and *Rhizoctonia solani* are among the most commonly associated with rot in fruits, vegetables, cereals, and ornamentals (Williamson et al., 2007; Boland and Hall, 1994; Bolton et al., 2006). These fungi thrive in moist environments and often infect through wounds or natural openings in plant tissues, leading to soft rot, dry rot, or stem and root decay, depending on the pathogen and host. Similarly, bacterial pathogens like *Pectobacterium* spp. and *Dickeya* spp. are known for causing soft rot diseases in vegetable crops, especially potato and ornamentals (Agrios, 2006; Charkowski et al., 2012; Charkowski, 2018; Arizala and Arif, 2024). These bacteria secrete plant cell wall-degrading enzymes (PCWDEs), such as pectinases and cellulases, that macerate plant tissues, resulting in water-soaked lesions and decaying tissue with foul odor (Charkowski et al., 2012; Hugouvieux-Cotte-Pattat et al., 2014; Charkowski, 2018; Arif et al., 2022).

Managing rot diseases is particularly challenging due to the diversity of causal organisms, the influence of environmental factors, and the lack of effective chemical controls for some pathogens (Dowling et al., 2020; Arif et al., 2022; Ma et al.). Cultural practices, breeding for disease resistance, and biological control agents are increasingly being integrated into management strategies to mitigate losses. Due to the ubiquitous nature and destructive potential of rot diseases, further research into pathogen biology, host resistance determinants, and integrated disease management methods is critical for sustainable crop production and food security.

In this Research Topic, nine articles were published. Ma et al. in their article “*Analysis of soft rot Pectobacteriaceae population diversity in US potato growing regions between 2015 and 2022*”, analyzes the population diversity of soft rot-causing *Pectobacteriaceae* in major U.S. potato-growing regions from 2015 to 2022. The samples were collected from commercial potato cropping systems from 14 states. Using whole genome sequencing and phenotypic profiling, they identified temporal and geographic variation among strains. Three species of *Dickeya* and eight species of *Pectobacterium* were detected in diseased potato samples. Among these, *Dickeya dianthicola, Pectobacterium parmentieri, P. carotovorum*, and *P. versatile* were the most prevalent, collectively accounting for 83% of the total isolates. Research article “*Retrospective survey of Dickeya fangzhongdai using a novel validated real-time PCR assay*” by Alic et al. developed a novel qPCR assay for the detection of *D. fangzhongdai* from infected tissues and water samples. The assay shows a selectivity of 100%, a diagnostic sensitivity of 76%, and a detection limit ranging from 311 to 2,275 cells/mL of plant extracts. The assay was used in a retrospective survey of selected host plants and environmental niches relevant to *D. fangzhongdai* in Europe. While *D. fangzhongdai* was not detected in any plant samples, 12% of surface water samples were found to be positive. The study highlights the importance of specific detection tools to prevent overlooking the pathogen or misidentifying it as general *Dickeya* spp. Article by Ahmad et al. entitled “*Characterization and toxicological potential of Alternaria alternata associated with post-harvest fruit rot of Prunus avium in China*” investigated the *A. alternata* fungus, which is associated with post-harvest fruit rot of *P. avium* (sweet cherry) in China. The research focuses on the characterization of the pathogen and its toxicological potential. The authors isolated *A. alternata* from infected fruit samples and conducted morphological and molecular analyses to confirm its identity. They also evaluated the pathogenicity of the isolates on cherry fruits and assessed the production of mycotoxins. The findings revealed that *A. alternata* is a significant post-harvest pathogen of *P. avium* in China, capable of producing mycotoxins that pose a potential risk to human health. The study highlights the importance of monitoring and managing *A. alternata* infections to ensure the safety and quality of cherry fruits. In the article “*Aabrm1-mediated melanin synthesis is essential to growth and development, stress adaption, and pathogenicity in Alternaria alternate*” by Li et al. investigated the role of the *Aabrm1* gene, which encodes scytalone dehydratase, a key enzyme in the DHN-melanin biosynthesis pathway in *A. alternata*. Deletion of *Aabrm1* resulted in significantly reduced melanin production, leading to the formation of orange-colored colonies with smooth spores. The mutant strains exhibited impaired development of infection structures and reduced penetration capabilities. The mutants also showed increased sensitivity to oxidative stress, with increased levels of hydrogen peroxide (H_2_O_2_) and superoxide anions (O_2_^−^). The virulence of the *Aabrm1* deletion mutants was reduced in non-wounded pear leaves but remained unchanged in wound-inoculated pear fruits. These findings underscore the critical role of *Aabrm1*-mediated melanin synthesis in the growth, stress adaptation, and pathogenicity of *A. alternata*. Yang et al. in the article “*Antagonistic effects of Talaromyces muroii TM28 against Fusarium crown rot of wheat caused by Fusarium pseudograminearum*“, evaluated the biocontrol potential of *T. muroii* strain TM28 against *F. pseudograminearum*, the causative agent of *Fusarium* crown rot (FCR) in wheat. The TM28 strain inhibited the mycelial growth of *F. pseudograminearum* by 87.8% within 72 h. Its cell-free fermentation filtrate exhibited strong antagonistic effects on both mycelial growth and conidial germination by compromising the pathogen's cell membrane integrity. Greenhouse experiments demonstrated that TM28 significantly enhanced wheat's fresh weight and height in the presence of the pathogen, bolstered antioxidant defense mechanisms, and reduced disease severity and pathogen abundance in the rhizosphere, roots, and stem bases. RNA sequencing of *F. pseudograminearum* under TM28′s influence revealed 2,823 differentially expressed genes, with notable downregulation in pathways related to cell wall and membrane synthesis. These findings suggest that *T. muroii* TM28 holds promise as a biocontrol agent against wheat FCR. Article entitled “*Rouxiella badensis, a new bacterial pathogen of onion causing bulb rot*”, by Zhao et al. identified *R. badensis* as a novel pathogen responsible for onion bulb rot in Georgia, USA. Seven strains were isolated from symptomatic onions during surveys conducted in 2014, 2020, and 2021. Genome analyses, including Average Nucleotide Identity (ANI) and digital DNA-DNA Hybridization (dDDH), confirmed these strains as *R. badensis*. While the strains did not induce symptoms on onion foliage, they caused bulb rot and necrotic lesions in red onion scale assays. Population studies revealed that *R. badensis* proliferated significantly within onion scales, reaching levels comparable to the known pathogen *Pantoea ananatis* PNA 97-1R. Core-genome analysis indicated that the onion-derived *R. badensis* strains formed a distinct group separate from strains isolated from other sources. This research marks the first report of *R. badensis* as a plant pathogen, highlighting its potential impact on onion cultivation. Research article “*Colletotrichum spp. diversity between leaf anthracnose and crown rot from the same strawberry plant*” by Hu et al. investigated the diversity of *Colletotrichum* species associated with leaf anthracnose (LA) and anthracnose crown rot (ACR) in strawberries. Sampling 100 strawberry plants from Zhejiang province, China, they isolated 309 *Colletotrichum* strains from both leaves and crowns. Morphological observations and phylogenetic analyses of multiple genes identified the isolates as part of the *C. gloeosporioides* species complex, predominantly *C. siamense*, with *C. fructicola* and *C. aenigma*. Pathogenicity tests revealed that isolates from leaves were more aggressive on strawberry leaves, while those from crowns were more virulent on crown tissues. These findings highlight the tissue-specificity and diversity of *Colletotrichum* species in strawberry anthracnose diseases. In an article titled “*Secretome analysis of Macrophomina phaseolina identifies an array of putative virulence factors responsible for charcoal rot disease in plants*,” Sinha et al. investigated the secretome of *M. phaseolina*, a necrotrophic fungal pathogen causing charcoal rot in over 500 plant species. Using liquid chromatography–electrospray ionization–tandem mass spectrometry, they identified 117 secreted proteins, including various virulence factors. Lignocellulolytic enzymes, such as xylanase, endoglucanase, and amylase, were detected and validated, highlighting their role in degrading plant cell walls. These findings enhance our understanding of the pathogenicity of *M. phaseolina*. Research article “*An efficient strategy combining immunoassays and molecular identification for the investigation of Fusarium infections in ear rot of maize in Guizhou Province, China*” by Shang et al. conducted a comprehensive analysis of *Fusarium* species associated with maize ear rot. They collected 175 samples from 76 counties and employed immunoassays alongside molecular identification techniques to detect and identify the *Fusarium* species present. The study revealed a diverse population structure of *Fusarium* species in the region, providing valuable insights into the epidemiology of maize ear rot. These findings are important for developing targeted disease management strategies for *Fusarium* ear rot on maize in Guizhou Province.

In conclusion, this Research Topic brings together a diverse collection of research articles that address critical aspects of rot-causing bacterial and fungal pathogens such as pathogenicity, taxonomy, pathogen identification, and the population genetics underlying pathogen diversity and evolution. Collectively, these contributions fill knowledge gaps, offering deeper insights into the mechanisms by which these pathogens infect crops and cause economically significant diseases. Improved knowledge in this area is vital for developing effective management strategies against soft rot diseases caused by bacteria and fungi, ultimately reducing agricultural losses associated with these devastating plant pathogens.
